# High-Quality Assembly and Analysis of the Complete Mitogenomes of German Chamomile (*Matricaria recutita*) and Roman Chamomile (*Chamaemelum nobile*)

**DOI:** 10.3390/genes15030301

**Published:** 2024-02-26

**Authors:** Jun Yang, Xinting Zhang, Zixuan Hua, Hongna Jia, Keke Li, Chengcheng Ling

**Affiliations:** College of Food and Bioengineering, Bengbu University, Bengbu 233030, China; 17856375053@163.com (X.Z.); 13695628646@163.com (Z.H.); 13155238531@163.com (H.J.); 18132834019@163.com (K.L.); lingchengcheng402@163.com (C.L.)

**Keywords:** *Matricaria chamomilla*, *Chamaemelum nobile*, mitochondrial genome, repetitive sequence analysis, phylogenetic analysis

## Abstract

German chamomile (*Matricaria chamomilla* L.) and Roman chamomile (*Chamaemelum nobile*) are the two well-known chamomile species from the Asteraceae family. Owing to their essential oils and higher medicinal value, these have been cultivated widely across Europe, Northwest Asia, North America, and Africa. Regarding medicinal applications, German chamomile is the most commonly utilized variety and is frequently recognized as the “star among medicinal species”. The insufficient availability of genomic resources may negatively impact the progression of chamomile industrialization. Chamomile’s mitochondrial genome is lacking in extensive empirical research. In this study, we achieved the successful sequencing and assembly of the complete mitochondrial genome of *M. chamomilla* and *C. nobile* for the first time. An analysis was conducted on codon usage, sequence repeats within the mitochondrial genome of *M. chamomilla* and *C. nobile*. The phylogenetic analysis revealed a consistent positioning of *M. chamomilla* and *C. nobile* branches within both mitochondrial and plastid-sequence-based phylogenetic trees. Furthermore, the phylogenetic analysis also showed a close relationship between *M. chamomilla* and *C. nobile* within the clade comprising species from the Asteraceae family. The results of our analyses provide valuable resources for evolutionary research and molecular barcoding in chamomile.

## 1. Introduction

German chamomile (*M. chamomilla* L.) and Roman chamomile (*C. nobile*) are the two well-known chamomile species from the Asteraceae family. Owing to their essential oils and higher medicinal value, these have been cultivated widely across Europe, Northwest Asia, North America, and Africa [1]. Regarding medicinal applications, German chamomile is the most commonly utilized variety and is frequently recognized as the “star among medicinal species”. Its volatile oil is frequently utilized in the realm of cosmetic care for its ability to alleviate inflammation, mitigate skin irritation, and diminish redness and swelling of the skin [2]. Moreover, it is widely consumed as an herbal tea, and it is easily accessible in the form of chamomile tea bags that contain flowers [3]. Several studies have reported two terpenoids such as bisabolol and chamazulene that have exhibited a range of beneficial properties including anti-inflammatory, antiallergic, antispasmodic, antibacterial, antipyretic, and antifungal effects [4,5]. Interestingly, in contrast to German chamomile, Roman chamomile is characterized as a perennial herb. This specific type of herbal tea has a long-standing history in folk medicine as a soothing remedy for an array of health conditions, encompassing headaches, colds, flu, stomach disorders, and gastrointestinal ailments [6]. Additionally, its essential oils derived from its flowers are widely utilized as ingredients in perfumes, alcoholic beverages, confectionery, desserts, and cosmetics [7]. Notably, the multi-purpose use of chamomile oils highlights their versatility and popularity in the fragrance and beauty industries [8]. To date, most reports about chamomile have focused on their traditional Chinese medicine value. However, there is limited information available on the organelle genomes of chamomile species. Investigating the mitogenomes of two chamomile species will offer a valuable genetic resource for future research endeavors.

The genomes of organelles play a vital role in maintaining the growth and development of organisms and various strategies are utilized to mitigate DNA damage in response to genotoxic stressors [9]. According to the NCBI database as of September 2023, the number of published plastomes is close to 13,000. Among these, angiosperms make up a significant majority (93.4%), with eudicots accounting for 68.4% and monocots for 20.7% [10]. Currently, the number of mitochondrial genomes is significantly lower compared to that of chloroplast genomes, likely due to the intricate structures observed in mitogenomes, which result from the accompanied reorganization of specific DNA fragments during violent redox reactions [11]. The mitochondrion is believed to have originated from an endosymbiotic relationship between an alphaproteobacterium and an archaeal-derived host cell based on the endosymbiosis theory [12]. Over time, this symbiotic association evolved, leading to the mitochondrion becoming a semi-autonomous organelle [13]. Mitochondria, which are commonly referred to as energy factories, have a significant role in a multitude of metabolic processes and are essential for energy production, synthesis, and degradation within living cells. Recently, mitochondria have been frequently utilized in studies aimed at elucidating evolutionary relationships and distinguishing between various species [14]. During the evolution, the chloroplast genome (cp) sequences underwent integration into the mitochondrial genome [15]. Horizontal gene transfer (HGT) is a significant factor in shaping the evolution of plant mitogenomes [16]. The extensive rearrangements observed in mitochondrial genomes could potentially be attributed to intracellular gene transfers. Relative synonymous codon usage (RSCU) is a measure of the relative likelihood of a particular codon compared to other synonymous codons that encode the same amino acid [17]. In addition, the field of genetic breeding has extensively employed mitogenomes [18,19]. For example, SSRs (Simple Sequence Repeats) and SNPs (Single Nucleotide Polymorphisms) have found widespread application in the rapid identification of plant species based on mitogenomes, particularly in the classification of Chinese herbal medicine [20,21]. These molecular markers offer a rapid and efficient means of species identification and can provide valuable insights into the genetic relationships among different plant species. However, the available literature of evolutionary patterns of mitogenomes within chamomile species is currently limited. As a result, the complete sequencing of the chamomile mitochondrial genomes has the potential to greatly benefit breeding endeavors and facilitate the development of novel cultivars.

The Asteraceae or Compositae, also referred to as the sunflower family, is the largest family of flowering plants. It comprises an extensive range of 23,000 species and is classified into 1620 genera and 12 subfamilies [22]. This family has a remarkable valuable as oil crops, horticultural resources, medicinal plants, invasive weeds, and highly cultivated plants for the cut flower industry [23]. These plants are distributed across different regions globally. In recent years, there has been a considerable amount of research conducted on the cp genomes of various plants within the Asteraceae family [24,25,26]. However, the study of mitochondrial (mt) genomes within this family remains relatively limited. Furthermore, no mitogenome in the chamomile species has been reported up to now. Here, we systematically analyzed the mitochondrial genome structures of two chamomile species, genomic repeats, relative synonymous codon usage, gene transfer, and the evolutionary relationships among the Asteraceae family, which will shed light on the genetic and evolutionary mechanisms within this family.

## 2. Materials and Methods

### 2.1. Plant Materials and Genome Sequencing

The two specimens used for testing, namely German chamomile and Roman chamomile, were initially obtained from Hefei City, Anhui Province (117.25° E, 31.86° N), which are currently preserved in Anhui Agricultural University (Hefei, China). The fresh leaves were carefully enveloped in aluminum foil and promptly frozen using flash nitrogen, ensuring their preservation at a temperature of −80 °C for future utilization. ADNA extraction kit (Tiangen Biotech, Co., Ltd., Beijing, China) was employed to extract total genomic DNA. The DNA library was prepared by utilizing the VAHTSTM Universal DNA Library Prep Kit. The short-paired reads were sequenced using the Illumina NovaSeq sequencer (150 bp paired-end). The gTube technology was employed to fragment genomic DNA into segments averaging around 10 kb in size. The DNA library was prepared by adding relevant reagents to the platform, and subsequently introduced into the Flowcell for real-time single-molecule sequencing using the PromethION sequencer to acquire raw sequencing data.

### 2.2. Mitogenome Assembly and Annotation

The SMARTdenovo software was utilized with default parameters to assemble mitogenomes [27,28]. To enhance the accuracy and quality of the mitogenomes, we employed minimap2 and pilon (v1.23) to polish the Illumina short-reads [29,30]. CPGAVAS2 (http://47.96.249.172:16019/analyzer/home, accessed on 2 November 2023) was used for mitochondria genome annotations with *Chrysanthemum indicum* as reference mitogenomes from GenBank:MH716014 [31]. The annotations were manually refined using the Apollo software version 1.11.8 [32]. The structures of the mitogenomes were visualized using OGDRAW software (v. 1.3.1) [33]. The two mitogenomes were deposited in GenBank with the accession numbers OR464823 and OR464824, respectively. The online tRNAscanSE service (http://lowelab.ucsc.edu/tRNAscan-SE/, accessed on 23 November 2023) was utilized to verify all transfer RNA genes [34].

### 2.3. Analysis of Repeat Sequences and the Transfer of DNA between Chloroplast and Mitochondrion

MISA-web55 (https://webblast.ipk-gatersleben.de/misa/ accessed on 25 November 2023), was employed to detect microsatellite sequence repeats. Tandem repeats were detected by applying the TRF software with the following default parameters. We used the REPuter web (https://bibserv.cebitec.uni-bielefeld.de/reputer/, accessed on 26 November 2023) with default parameters to detect dispersed repeats [35]. We conducted a sequence similarity analysis between the cp genomes and the mitogenomes using the BLASTN tool to identify transferred DNA fragments and used the Circos package in TBtools to visualize the results [36,37].

### 2.4. Codon Usage and Phylogenetic Inference Analyses

The estimation of nucleotide composition and relative synonymous codon usage (RSCU) was performed using MEGA v.7 [38]. A total of 12 mitochondrial genomes of Asteraceae were retrieved from GenBank to construct phylogenetic tree. A phylogenetic tree was performed using IQTREE based on the cp genome and mitogenome of two chamomile species and 9 other species that are closely related and was generated using the maximum likelihood (ML) method in RAxML v8.1.5, with 1000 bootstrap replicates. The phylogenetic trees were displayed using the online tool iTOL (https://itol.embl.de, accessed on 29 November 2023).

## 3. Results

### 3.1. Mitogenome Assembly, Annotation, and Gene Features

**Figure 1:**
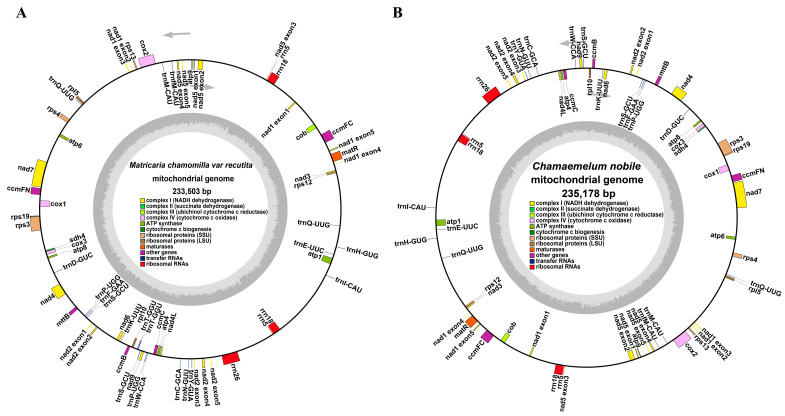
The circular maps of mitogenomes. (**A**) The circular diagram illustrating the mitochondrial genome of *M. chamomilla*. (**B**) The circular diagram illustrating the mitochondrial genome of *C. nobile*. The genomic characteristics transcribed in the clockwise and counterclockwise directions are depicted on the inner and outer regions of the circular map, respectively. The functional classification of genes in the mitochondrial genome is visually represented by color-coding. The GC content is visually represented in the inner circle using a dark gray plot.

We successfully assembled a single circular molecule for two chamomile species, with sizes of 233,503 kb and 235,178 kb for *M. chamomilla* and *C. nobile*, respectively (Figure 1A,B). The nucleotide composition analysis results of the complete *M. chamomilla* mitogenome (45.11% GC content) showed the following percentages—A, 27.60%; T, 27.29%; C, 22.32%; and G, 22.79%—which are similar to those of *C. nobile* (A, 27.65%; T, 27.27%; C, 22.31%; and G, 22.77%, 45.08% GC content). The *M. chamomilla* mitochondrial genome has 57 genes (51 unique genes), including 32 protein-coding genes (32 are unique), 20 tRNA genes (16 are unique), and 5 rRNA genes (3 are unique) (Figure 1; Table S1). The genes rrn5 and rrn18 exhibit a duplication event in the mitochondrial genome, resulting in the presence of two copies for each gene. The *C. nobile* mitochondrial genome has 54 genes (50 unique genes), including 32 protein-coding genes (32 are unique), 17 tRNA genes (15 are unique), and 5 rRNA genes (3 are unique) (Figure 1A; Table S1). The variable genes rrn5 and rrn18 have two copies in the mitochondrial genome (Figure 1B; Table S2). 

### 3.2. Analysis of Repeat Sequences

**Figure 2:**
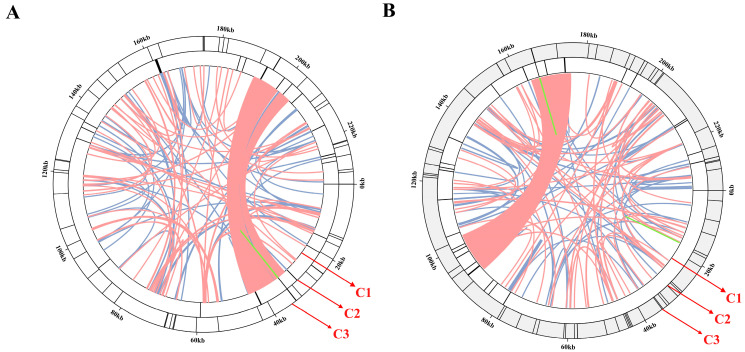
The repeats analysis of the *M. chamomilla* and *C. nobile* chamomile mitochondrial genomes. (**A**) The repeat sequences detected in the mitochondrial genome of *M. chamomilla*. (**B**) The repeat sequences detected in the mitochondrial genome of *C. nobile*. The dispersed repeats, indicated by green, blue, or pink arcs, are represented in the C1 circle. Tandem repeats are depicted as short bars in the C2 circle. The C3 circle illustrates the microsatellite sequences detected.

A total of 57 and 65 Simple Sequence Repeats (SSRs) were identified in the mitogenome of *M. chamomilla* and *C. nobile*, respectively (Figure 2A,B, Tables S4 and S5). The distribution of SSRs, including mono-, di-, tri-, tetra-, or pentanucleotide, is evenly spread among the various types in the mitochondrial genome of two chamomile species. However, only two hexamers were discovered in *C. nobile* (Table S5). Tetranucleotide repeat units were the most prevalent type of SSRs in the *M. chamomilla* and *C. nobile* mitochondrial genomes, accounting for 41.3% and 33.8% of all repeat numbers, respectively (Tables S4 and S5). Tandem repeat sequences are a ubiquitous feature among the genomes of all organisms that have been sequenced. We identified 28 and 31 tandems in the *M. chamomilla* and *C. nobile* mitochondrial genomes, respectively (Tables S6 and S7). Further evaluation was conducted on these repeats to determine their suitability as markers for DNA fingerprinting purposes. The dispersed repetitive sequence was categorized into four types: direct, reverse, complement, and palindromic (inverted) repeats. In the *C. nobile* mitochondrial genome, all four types of dispersed repeats were detected (Tables S8 and S9). However, three types of dispersed repeats, including direct, reverse, and palindromic (inverted) repeats, were detected in the *M. chamomilla* mitochondrial genome. The dispersed repeats of the *M. chamomilla* and *C. nobile* mitochondrial genomes were arranged in ascending order according to their e-values.

### 3.3. Analysis of Homologous Sequences between Two Organelles

**Figure 3:**
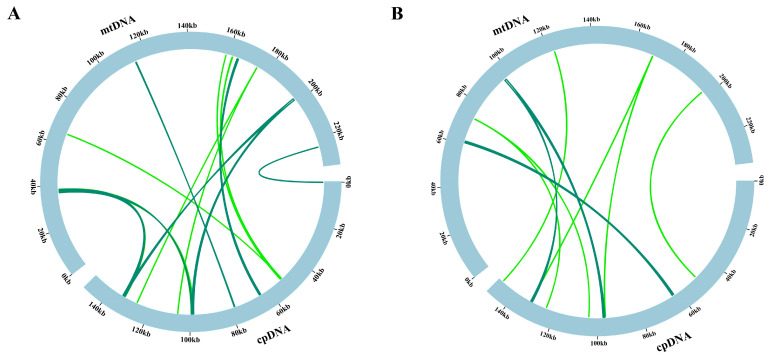
The homologous DNA sequences between the chloroplast genome and mitogenome of *M. chamomilla* and *C. nobile*. (**A**) The conserved DNA sequences shared between the chloroplast and mitochondrial genomes of *M. chamomilla*. (**B**) The conserved DNA sequences shared between the chloroplast and mitochondrial genomes of *C. nobile*. The inner green arcs illustrate the homologous DNA fragments. The visualization of homologous sequences between the organelle genomes was performed using the TBtools software (v2.012) with incorporation of the Circos package.

The translocation of mitochondrial and plastid DNAs to the nucleus is recognized as a crucial component of genome evolution and exerts a substantial influence on the evolution of eukaryotes [39,40]. In the mitogenome of *M. chamomilla* and *C. nobile*, we found that the total lengths of 4763 and 3260 base pairs (bp) of sequences likely originated from the corresponding cp genome (Figure 3A,B; Tables S10 and S11). These homologous sequences accounted for approximately 2.03% and 1.38% of the respective individual mitogenomes.

### 3.4. Analysis of Codon Usage PCGs

**Figure 4:**
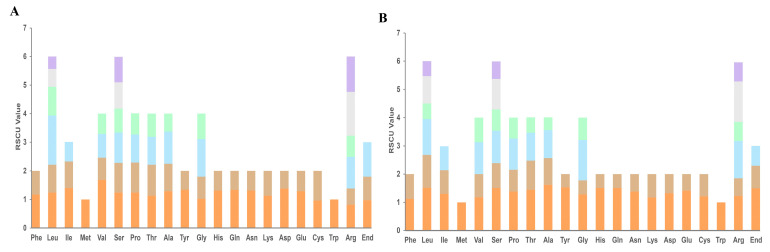
Relative synonymous codon usage (RSCU) in mitochondrial protein-coding genes in the mitogenome of *M. chamomilla* and *C. nobile*. The value for RSCU is represented on the y-axis. (**A**) The RSCU value of *M. chamomilla*. (**B**) The RSCU value of *C. nobile*.

An analysis of codon distribution and relative synonymous codon usage (RSCU) was conducted on the mitogenomes of two chamomile species. The analysis of RSCU indicated that Leucine was the most prevalent amino acid. among the two chamomile species mitogenomes, whereas codons encoding Met were the least abundant, which is very common in mitogenomes of land plant species. The 57 and 54 annotated protein-coding genes are encoded by codons 9322 in German chamomile and 9322 in Roman chamomile. Two chamomile species exhibited a similar RSCU style pattern and the majority of protein-coding genes (PCGs) in these species initiated with the standard ATG start codon (Figure 4).

### 3.5. Phylogenetic Tree Analysis

**Figure 5:**
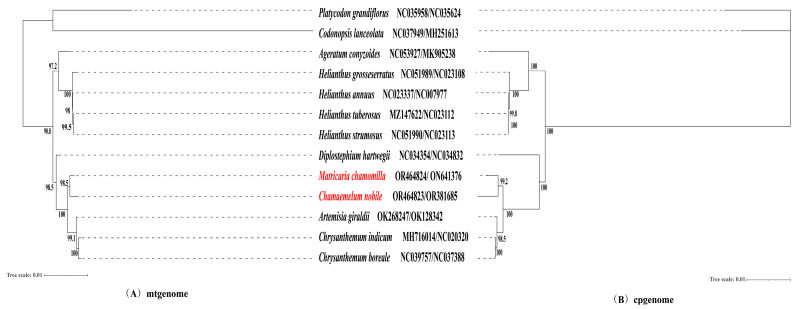
The maximum likelihood (ML) analysis was utilized to investigate the phylogenetic relationships among *M. chamomilla*, *C. nobile*, and nine other species belonging to the Asteraceae family. The sequence obtained in this study has been distinguished by highlighting it in red. The GenBank accession numbers for the chloroplast genomes and mitochondrial genomes are provided next to the corresponding Latin names of the species.

In order to enhance our understanding of the evolutionary relationships within the Asteraceae family, we selected 11 organelle genomes from species within the Asteraceae family for analysis and *Platycodon grandiflorus* and *Codonopsis lanceolata* as outgroups from GenBank. The cpgenomes and mtgenomes were subsequently utilized to construct phylogenetic trees based on 64 PCGs and 17 PCGs, respectively. Both phylogenetic trees revealed a division of the 13 species into two main clades. The larger clade consisted of 11 species from the Asteraceae family, while the smaller clade consisted of two outgroup species (Figure 5). Additionally, the phylogenetic analysis indicated a close relationship between *M. chamomilla* and *C. nobile* within the Asteraceae clade (Figure 5).

## 4. Discussion

*M. chamomilla* and *C. nobile*, also referred to as “stars among medicinal species”, have long been recognized for their traditional medicinal value. Acquiring their genomic information is a crucial step in comprehending the biosynthesis of its active substance. Here, we performed the sequencing and assembly of the complete mitochondrial genomes of two chamomile species for the first time. The two mitochondrial genome assemblies were conducted using a graph-based approach, integrating NGS reads with long reads. This approach ensures a more accurate and coherent integration of NGS data, thereby enhancing the overall effectiveness of the assembly process [41]. Consequently, the resulting findings are more robust and can be confidently relied upon for further analysis and interpretation. In this study, a single circular molecule has been identified in the complete mitochondrial genomes of two chamomile species, respectively. This phenomenon has been found in the most Asteraceae mitogenomes [42]. Wang et al. reported that the variation in mitogenome sizes within the Asteraceae family is highly variable from 186,772 to 356,991 [43]. The mitogenome size of *M. chamomilla* and *C. nobile* is 233,503 kb and 235,178 kb, respectively. The size of their mitogenomes varies within the Asteraceae family. The variation in mitogenome size within the Asteraceae family can be attributed to several factors, including the presence of significant repetitive and foreign fragments [44,45].

In contrast to the conserved monocyclic structure typically observed in chloroplast genomes, the mitochondrial genomes of seed plants often display multiple alternative conformations or minor variations attributed to the existence of repeat sequences [46,47]. Repeated sequences, including tandem repeats, SSRs, and dispersed repeats, are abundant in plant mitochondrial genomes according to research findings [48]. SSRs play a crucial role as molecular markers in various areas of study, such as species identification, evolutionary analysis, and genetic diversity exploration [49]. Tandemly repeated DNA sequences with unit lengths exceeding six base pairs display notable genomic variability as a result of their propensity for gaining or losing repeat units [50]. Previous studies have indicated that nearly all angiosperm mitochondrial genomes exhibit substantial non-tandem repeats, typically exceeding 1 kb in length, and these repeats are actively involved in recombination events [51]. One possible explanation for isomerization in two chamomile mitochondrial genomes could be the presence of the longest non-tandem repeat, which spans a range of 78,982–22,6742 base pairs. Among the dispersed repeats in German chamomile and Roman chamomile mitochondrial genomes, palindromic repeats were found to be the most abundant. This finding was also reported in the *Artemisia giraldii* [52]. Dispersed repeats play a crucial role in fostering genetic diversity and are instrumental in shaping the evolution of plant genomes [53].

Gene transfer from the chloroplast to the mitogenome is a common occurrence throughout the long-term evolution of plants [54]. Mitochondrial genomes frequently contain plastid-DNA-derived sequences known as mitochondrial plastid DNAs (MTPTs) [55,56]. There is limited evidence of DNA transfer between organellar genomes within the Asteraceae family reported in the literature. BLAST search results indicated that the inserted sequences showed a high degree of similarity to mitochondrial DNA sequences from *Chrysanthemum*, *Diplostephium*, *Lactuca*, *Helianthus*, and *Paraprenanthes* [42]. In this study, we conducted an analysis of the homologous sequences between the mitogenomes and plastomes of two chamomile species. The combined length of the nineteen fragments was 4782 base pairs (bp), representing 2.04% of the entire Roman chamomile mitogenome. The combined length of the nineteen fragments was 3269 base pairs (bp), representing 1.39% of the entire Roman chamomile mitogenome (Figure 3). Yue et al. reported similar results in soybean [52]. The extensive rearrangements observed in mitochondrial genomes could potentially be attributed to intracellular gene transfers [57]. Therefore, we speculated that the fragmentation of mitochondrial genomes could be attributed to the integration of segments from the chloroplast genome, which displayed a high level of alignment with the original chloroplast genome sequences during gene transfer events. Among the amino acids analyzed, Leucine (Leu), Serine (Ser), and Arginine (Arg) were found to be the most frequent, while Tryptophan (Trp) and Methionine (Met) were identified as the least common (Figure 4), which is frequently observed in the genomes of plant mitochondria [46,58,59]. Although two chamomile species can be distinguished by morphological characteristics (for instance, one is perennial and the other annual), there is a limited amount of existing literature that explores the evolutionary relationship between the two chamomile species. We employed the sequences of conserved genes to generate phylogenetic trees for mitochondria and plastids using maximum likelihood (ML) methods. The results suggested that the sequence conservation among ten Asteraceae species corresponded with the clustering observed in the mitochondrial phylogenetic analysis [52]. Our result also showed the same relationships between 10 Asteraceae species. Compared to the four Helianthus species and *A. conyzoides*, *A. giraldii* exhibited higher sequence similarity with two chamomile species. The phylogenetic trees showed a close relationship between the *M. chamomilla* and *C. nobile* in Asteraceae (Figure 5), which is consistent with the result reported by Tai et al. [5]. The information has the potential to contribute significantly to the understanding of phylogenetics and evolutionary processes within chamomile species.

## 5. Conclusions

In our study, we successfully assembled the mitogenomes of *M. chamomilla* and *C. nobile* for the first time. The mitogenome size of *M. chamomilla* and *C. nobile* is 233,503 kb and 235,178 kb, respectively. The size of their mitogenomes varies within the Asteraceae family. The phylogenetic analysis demonstrated concordance in the branch positions of *M. chamomilla* and *C. nobile* within the phylogenetic trees constructed using both mitochondrial and plastid sequences. Moreover, the phylogenetic analysis revealed a close relationship between *M. chamomilla* and *C. nobile* within the clade comprising species from the Asteraceae family. Our findings have contributed valuable mitochondrial genome resources for the Asteraceae family, thereby enhancing our comprehension of organelle genome evolution in flowering plants.

## Data Availability

The study’s accession number can be found in OR464823 for *M. chamomilla* and OR464824 for *C. nobile*. The raw sequencing data (*M. chamomilla* and *C. nobile*) have been deposited in NCBI and can be accessed using the following accession numbers: PRJNA1070027, SAMN39634864, SAMN39634865, SRR27751387, and SRR27751388.

## Supplementary Materials

The following supporting information can be downloaded at https://www.mdpi.com/article/10.3390/genes15030301/s1. Table S1. Gene composition in the mitogenome of *M. chamomilla.* Table S2. Gene composition in the mitogenome of *C. nobile.* Table S3. Features of two mitogenomes belonging to the chamomile. Table S4. Microsatellite repeats in the *M. chamomilla* mitogenome. Table S5. Microsatellite repeats in the *C. nobile* mitogenome. Table S6. Tandem repeats in the *M. chamomilla* mitogenome. Table S7. Tandem repeats in the *C. nobile* mitogenome. Table S8. Dispersed repeats in the *M. chamomilla* mitogenome. Table S9. Dispersed repeats in the *C. nobile* mitogenome. Table S10. DNA transfer length of *M. chamomilla* organelle genomes. Table S11. DNA transfer length of *C. nobile* organelle genomes.
